# Mechanical, Cardiorespiratory, and Muscular Oxygenation Responses to Sprint Interval Exercises Under Different Hypoxic Conditions in Healthy Moderately Trained Men

**DOI:** 10.3389/fphys.2021.773950

**Published:** 2021-12-16

**Authors:** Robert Solsona, Hugues Berthelot, Fabio Borrani, Anthony M. J. Sanchez

**Affiliations:** ^1^University of Perpignan Via Domitia (UPVD), Faculty of Sports Sciences, Laboratoire Interdisciplinaire Performance Santé Environnement de Montagne (LIPSEM), UR4640, Perpignan, France; ^2^Institute of Sport Sciences, University of Lausanne, Lausanne, Switzerland

**Keywords:** blood flow restriction (BFR), exhaustive exercise, hypoxia, oxygen extraction, skeletal muscle, supine exercise, gravity-induced blood flow restriction, vascular occlusion

## Abstract

**Objective:** The aim of this study was to determine the effects of sprint interval exercises (SIT) conducted under different conditions (hypoxia and blood flow restriction [BFR]) on mechanical, cardiorespiratory, and muscular O_2_ extraction responses.

**Methods:** For this purpose, 13 healthy moderately trained men completed five bouts of 30 s all-out exercises interspaced by 4 min resting periods with lower limb bilateral BFR at 60% of the femoral artery occlusive pressure (BFR_60_) during the first 2 min of recovery, with gravity-induced BFR (pedaling in supine position; G-BFR), in a hypoxic chamber (FiO_2_≈13%; HYP) or without additional stress (NOR). Peak and average power, time to achieve peak power, rating of perceived exertion (RPE), and a fatigue index (FI) were analyzed. Gas exchanges and muscular oxygenation were measured by metabolic cart and NIRS, respectively. Heart rate (HR) and peripheral oxygen saturation (SpO_2_) were continuously recorded.

**Results:** Regarding mechanical responses, peak and average power decreased after each sprint (*p* < 0.001) excepting between sprints four and five. Time to reach peak power increased between the three first sprints and sprint number five (*p* < 0.001). RPE increased throughout the exercises (*p* < 0.001). Of note, peak and average power, time to achieve peak power and RPE were lower in G-BFR (*p* < 0.001). Results also showed that SpO_2_ decreased in the last sprints for all the conditions and was lower for HYP (*p* < 0.001). In addition, Δ[O_2_Hb] increased in the last two sprints (*p* < 0.001). Concerning cardiorespiratory parameters, BFR_60_ application induced a decrease in gas exchange rates, which increased after its release compared to the other conditions (*p* < 0.001). Moreover, muscle blood concentration was higher for BFR_60_ (*p* < 0.001). Importantly, average and peak oxygen consumption and muscular oxyhemoglobin availability during sprints decreased for HYP (*p* < 0.001). Finally, the tissue saturation index was lower in G-BFR.

**Conclusions:** Thus, SIT associated with G-BFR displayed lower mechanical, cardiorespiratory responses, and skeletal muscle oxygenation than the other conditions. Exercise with BFR_60_ promotes higher blood accumulation within working muscles, suggesting that BFR_60_ may additionally affect cellular stress. In addition, HYP and G-BFR induced local hypoxia with higher levels for G-BFR when considering both exercise bouts and recovery periods.

## Introduction

Physical exercise promotes the modulation of a large panel of cellular signaling pathways to promote metabolic and/or morphological changes that enhance performance (Bishop et al., 2019; Sanchez et al., 2019; Solsona et al., 2021). In recent years, repetition of short (≤30 s) all-out exercises were boosted in popularity because they can induce similar or higher gains of performance for a lower training volume compared to conventional endurance protocols (MacDougall et al., 1998; Barnett et al., 2004; Burgomaster et al., 2006, 2008; Gibala et al., 2006). Two distinct repeated sprint protocols are distinguished. Repeated sprint training (RST) consists of maximal sprint exercises of short duration (≤10 s) interspaced with short recovery periods (i.e., exercise to rest ratio <1:4). On the other hand, sprint interval training (SIT) includes repeated long sprints (~30 s) interspaced by longer rest periods (~2–4 min) (Buchheit and Laursen, 2013a,b; Brocherie et al., 2017). In recent years, studies have suggested that the addition of a hypoxic stimulus to chronic SIT and RST may favor several training responses (Faiss et al., 2013; Brocherie et al., 2017; Brechbuhl et al., 2020; James and Girard, 2020). For example, systemic hypoxia (HYP) may promote higher muscle perfusion and oxygenation with greater modulations of molecular adaptations (Faiss et al., 2013; Brocherie et al., 2017). These changes include increases in HIF-1α, myoglobin, and the expression of genes involved in mitochondrial biogenesis.

In addition to HYP, blood flow restriction (BFR) training strategies can also favor several training adaptations (Preobrazenski et al., 2021). BFR results in reduced arterial and/or venous blood flow according to the level of pressure exerted, thus generating local hypoxia and an accumulation of metabolites in the working muscles (Sugaya et al., 2011; Teixeira et al., 2018; Okita et al., 2019). BFR exercise training was found to be beneficial for enhancing adaptations such as muscle hypertrophy and aerobic capacity (Preobrazenski et al., 2021). Acute exercise with BFR may increase neuromuscular activation and type II muscle fibers recruitment (Moritani et al., 1992). In addition, BFR aerobic exercise stimulates ventilatory and cardiac response through the stimulation of group III and IV afferents (Adreani and Kaufman, 1998). These acute responses result in increased energy expenditure leading to a loss of muscle efficiency during submaximal exercise (Ozaki et al., 2010; Mendonca et al., 2014; Silva et al., 2019). However, to the best of our knowledge, BFR studies were conducted with low-intensity exercises or during RST (Willis et al., 2019), and little is known about SIT protocols using BFR.

Furthermore, different BFR models, such as cuff-, pressure chamber-, and gravity-models rise in popularity, and studies are needed to highlight their specific effects and related adaptations. Recently, a gravity-induced BFR (G-BFR) aerobic protocol has been investigated in a study conducted by Preobrazenski and colleagues (Preobrazenski et al., 2020). An ergocycle titled 45° was used to generate ischemia within working muscles. In this study, muscle oxygenation was effectively found to be reduced during submaximal aerobic exercises, as previously indicated during supine exercise where faster O_2_ uptake kinetics was also observed (Hughson et al., 1991, 1993). Of note, Preobrazenski et al. suggested that the G-BFR model seems favorable to enhance aerobic adaptations (Preobrazenski et al., 2020). However, nothing is known about the use of such a protocol during SIT. Importantly, to our knowledge, the acute effects of HYP and BFR models on both cardiovascular and muscular responses have never been compared during SIT protocols. It appears important to understand the acute effects of these models to identify potential training adaptations for the efficient prescription of training strategies.

Thus, the goal of this study was to assess the effects of each condition on mechanical output, cardiorespiratory responses, and muscle oxygenation in healthy moderately trained men. In the present study, it has been chosen to set maximal stress for both HYP and BFR conditions that could be tolerated in this cohort in combination with the SIT protocol proposed based on preliminary work in our laboratory. Concerning the G-BFR condition, the inclined position was maintained during the entire exercise session. The hypotheses were that we would observe different responses depending on the condition with (i) a higher power output during the successive exercise bouts in NOR since the other conditions induce additional stress that may lead to a reduction of acute muscle efficiency and subsequent performance, (ii) a greater impact of HYP on the cardiorespiratory responses because HYP causes hypoxemia, and (iii) a more pronounced impact of BFR models on muscle oxygenation because they generate local hypoxia.

## Materials and Methods

### Subjects

Thirteen healthy moderately trained men (mean ± SD, age 24 ± 3 years; weight 73.8 ± 6.5 kg; height 179 ± 6 cm; body fat percentage 12.5 ± 2.1%; training frequency 8 ± 4 h per week) participants took part in this experiment. Prior to the first visit, the participants were informed about the experimental procedures and the possible discomforts and risks. The participants provided written informed consent and completed a questionnaire to exclude all potential cardiorespiratory and injury risks. The experimental protocol was approved by the local ethics committee (VD-2021-00597). All experiments were performed in accordance with the last Declaration of Helsinki.

### Study Design

Participants visited the laboratory on four occasions over 4 weeks. During the 4 days of experimentation, participants performed an SIT session in different conditions, namely normal condition (NOR), with bilateral limb blood flow restriction at 60% of the total femoral artery pressure (BFR_60_) during the first 2 min of recovery (Taylor et al., 2016; Mitchell et al., 2019), with G-BFR, and in a hypoxic room at FiO_2_≈13% (HYP). Exercises were performed in a random order established by an independent blinded researcher. Sessions lasted between 90 and 120 min and were separated by at least 5 days to avoid fatigue-related interferences with the exercise sessions. Anthropometric measurements were carried out on the first visit and body fat percentage was estimated with the four skinfold thickness method (Durnin and Womersley, 1974). All tests were performed at the same time of the day to minimize the effects of circadian cycles and within similar environmental conditions. The participants were asked to maintain their dietary habits without alcohol consumption 48 h before each test. Athletes did not take medication or dietary supplements during the studied period. A standardized diet (55% carbohydrate, 15% protein, and 30% fat) was proposed to the participants the day preceding each test.

### Determination of Femoral Artery Occlusion Pressure

Total femoral artery occlusion pressure was measured on the day the participant followed the BFR protocol to avoid a potential effect of time and to be more accurate. The participants sat on a chair for the measurement of the total femoral artery occlusion pressure. The cuffs (SC12D, cuff size 13 cm × 85 cm) were placed around the right inferior limb proximal to the hip articulation. The occlusion pressure was progressively increased with the inflation apparatus (E20/AG101 Rapid Cuff Inflation System, D.E Hokanson Inc., Bellevue, WA, United States). The occlusion level was determined with an ultrasound linear probe (EchoWave II 3.4.4, Telemed Medical Systems, Milan, Italy) to measure blood flow. Total occlusion pressure was considered as reached when there was no detectable arterial blood flow. A total of three measurements were taken with a 1 min recovery between each evaluation. The highest-pressure value obtained was used to determine the 60% pressure applied for BFR_60_ during the exercise sessions. Importantly, the cuff pressure of 60% was used following preliminary work performed in the laboratory that highlighted it was the highest level that could be tolerated by the participants in combination with the present SIT protocol.

### Exercise Sessions

All testing was performed in a controlled indoor environment with an ergocycle (Lode Excalibur Sport 911905, Lode B.V., Groningen, The Netherlands) programmed on constant torque mode (Wingate mode) with a torque factor of 0.8 Nm.kg^−1^. The warm-up consisted of 10 min of cycling at 100 W (85 rpm) and two 6-s maximal sprints interspaced by a passive recovery of 54 s. Then, after 4 min recovery, the participants completed five bouts of 30 s standing start all-out exercises interspaced by 4 min rest periods. BFR was applied with inflatable cuffs during the first 2 min of recovery after each sprint. Concerning the G-BFR condition, participants maintained their inclined position during exercise bouts and recovery periods. For this condition, a structure was built to allow participants to lay horizontally on their backs as comfortably as possible. The structure also permitted handgrip to avoid body displacements during exercise. HYP was normobaric and was used during the whole session, such as the warm-up. NOR was performed below 400 m of altitude. The configuration (height and length) of both the saddle and handlebars was recorded to be reproduced in subsequent tests. Participants had to maintain saddle contact. They were encouraged energetically to complete every exercise maximally. Verbal indication of time was not provided to minimize pacing strategies during each sprint exercise.

Participants quoted their subjective perception of effort through the 6–20 rating perceived exertion (RPE) scale after each sprint. Individual measurements of peak (*P*_*peak*_), minimal (*P*_min_), and average power, time to achieve peak power, and a fatigue index (FI) were collected. FI was calculated as follow for each sprint:

$$\begin{array}{l} FI=\frac{P_{peak}-P_{\min}}{P_{peak}}\;\times 100 \end{array}$$

During the BFR_60_ condition, the cuffs were placed bilaterally and proximally to the hip articulation. The cuffs were inflated during the first 2 min of recovery. The laboratory where the tests took place was below 400 m of altitude.

### Gas Exchanges and Peripheral Oxygen Saturation Measurements

Breath-by-breath gas exchanges and peripheral oxygen saturation (SpO_2_) were continuously monitored throughout the exercises and recovery periods. Oxygen consumption ($\dot{\text{V}}$O_2_), carbon dioxide ($\dot{\text{V}}$CO_2_) production, and minute ventilation ($\dot{\text{V}}$E) were measured with a gas exchange analyzer (Quark CPET, COSMED, Rome, Italy). Tidal and gas volumes (ΣVT, ΣVO_2_, and ΣVCO_2_) were cumulated for each period (exercise and recoveries). Data were treated using a second-order Butterworth filter with a cutting frequency of 0.1 Hz. Breathing flow was measured by a bi-directional digital turbine that was calibrated using a 3-l syringe (C00600-01-11, Cosmed, Rome, Italy). A known gas mixture (O_2_: 15.05%, CO_2_: 5.05%) was used to calibrate O_2_ and CO_2_ analyzers. Heart rate was collected with a Garmin monitor (HRM3-SS, Garmin, Southampton, United Kingdom). Peak and minimal values were determined for these variables during each sprint. Delta values (Δ) were calculated as the absolute difference between peak and minimal values. SpO_2_ was continuously recorded with a pulse oximeter (WristOx 3100, Nonin Medical Inc., Amsterdam, The Netherlands) and the sensor (8000Q2Sensor, Nonin Medical Inc., Amsterdam, The Netherlands) was placed at the earlobe.

### NIRS Measurements and Data Assessment

Muscular O_2_ extraction measurements were monitored by an absolute near-infrared spectroscopy (NIRS) probe (OxiplexTS, ISS, Champagne, USA). The device was placed on the distal portion of the right vastus lateralis and was held by an elastic band wrapped around it to minimize extraneous light and movement. NIRS device includes four transmitters situated at 2.5, 3.0, 3.5, and 4.0 cm from the receptor. The acquisition frequency was 50 Hz and data were averaged every 1 s. Two different wavelength laser diodes provided the light source (682 and 834 nm), and the differential pathlength factor was set to 4. Oxygen extraction was estimated by the tissue saturation index (TSI) from the NIRS measurement, which also includes total hemoglobin concentration ([tHb]), and concentrations of deoxyhemoglobin ([HHb]), and oxyhemoglobin ([O_2_Hb]).

### Statistical Analysis

The statistical analyses were performed using Jamovi (Version 1.6.15.0). After inspecting residual plots, no obvious deviations from homoscedasticity or normality were observed. Therefore, linear mixed models were used to be more accurate with the specificity of our experimental design. Indeed, this is a longitudinal approach and linear mixed models (LMM) use mixed effect modeling to provide more precise estimates when data are hierarchized compared to repeated measures ANOVA (Gueorguieva and Krystal, 2004; Boisgontier and Cheval, 2016; Muth et al., 2016). Indeed, LMM have been developed to consider both the nested (i.e., multiple observations within a single participant in a given condition) and crossed structure (i.e., participants observed in multiple situations) of the data (Baayen et al., 2008; Boisgontier and Cheval, 2016). The flexibility of LMM makes them more appropriate for analyses of repeated measures data and when working with missing data or limited samples (Gueorguieva and Krystal, 2004; Boisgontier and Cheval, 2016; Muth et al., 2016). Conditions (i.e., NOR, BFR_60_, G-BFR, and HYP) and sprint number were the fixed effects, and the participant was set as the random effect. *Post-hoc* comparisons with Holm's corrections for multiple comparisons were used to adjust *p*-values. The level of significance was set at 0.05 and dispersion about the mean was expressed as SD. Effect sizes (d) (Judd et al., 2017) are provided (trivial effect *d* < 0.10, small effect 0.10 ≤ d < 0.50, medium effect 0.50 ≤ d < 0.80 and large effect d ≥ 0.80).

## Results

No interaction between conditions and sprint number was found for any variable (*p* > 0.05).

### BFR Pressure, RPE, and SpO_2_

**Figure 1:**
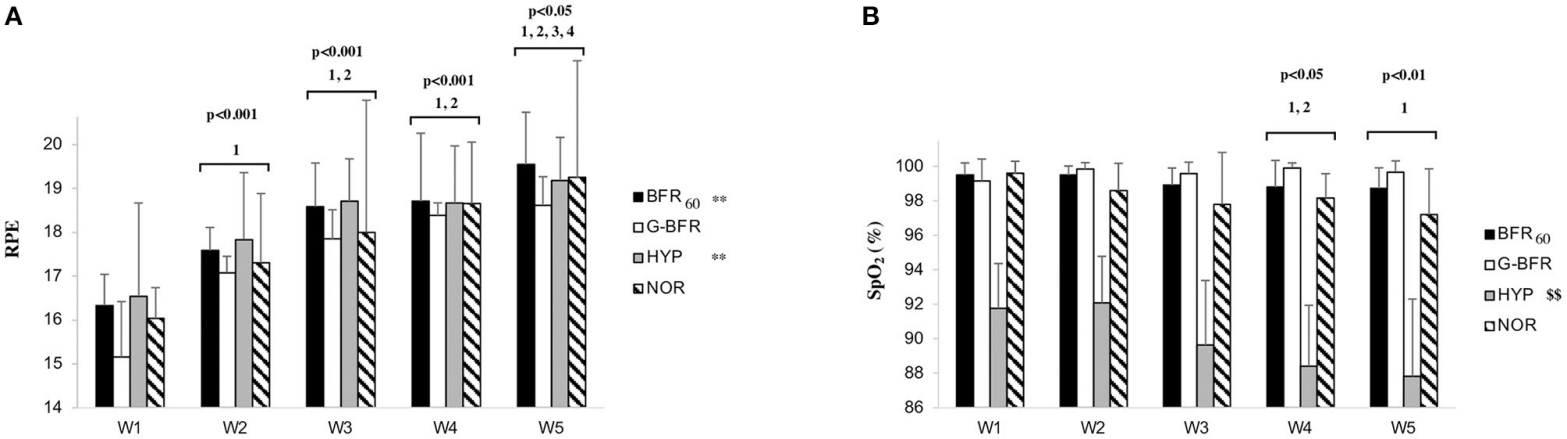
Rating of perceived exertion (RPE) **(A)** and SpO_2_ **(B)** values between the different conditions. BFR_60_, blood flow restriction during the two first minutes of recovery; HYP, hypoxia in a chamber at 13.0% FiO_2_; G-BFR, gravity-induced BFR; NOR, control. Differences between the numbers of the sprint are highlighted by numbers and symbols are used for differences between conditions. ^1^Different from sprint number one; ^2^different from the second sprint; ^3^different from the third sprint; ^4^different from the fourth sprint, these differences concern the effect of sprint number without distinguishing the exercise modality (n.b., no statistical interaction was found); **p < 0.01 different from G-BFR; ^$$^*p* < 0.01 different from the other conditions.

The total occlusion pressure of the participants was 191.0 ± 13.9 mmHg and BFR_60_ pressure was 114.6 ± 8.3 mmHg. A significant main effect of the condition (*p* < 0.01) and sprint number (*p* < 0.01) was found on RPE values, with higher values observed for BFR_60_ and HYP compared to G-BFR (*p* < 0.01, *d* = 0.2 for both). No difference was found between NOR and the other conditions for RPE. RPE increased significantly throughout the sprints (*p* < 0.05), except between sprints three and four (*p* > 0.05) (Figure 1A). Concerning the average SpO_2_, a main effect of the condition was found (*p* < 0.01). *Post-hoc* analyzes revealed that SpO_2_ was lower for HYP compared to the other conditions (*p* < 0.01, d = −2.1, −2.5, and −1.9 for BFR_60_, G-BFR, and NOR, respectively). The values were 91 ± 5, 99 ± 1, 100 ± 1, and 98 ± 2% for HYP, BFR_60_, G-BFR, and NOR, respectively. Moreover, there was a main effect of the sprint number on SpO_2_ (*p* < 0.01). SpO_2_ significantly decreased (*p* < 0.05) between the first and the last sprint, and between the second and the last two sprints (Figure 1B). Remarkably, BFR_60_ did not induce a reduction of SpO_2_ during its application compared to the other conditions (*p* > 0.05).

### Peak and Average Power Output, Time to Achieve Peak Power, and Fatigue Index

**Figure 2:**
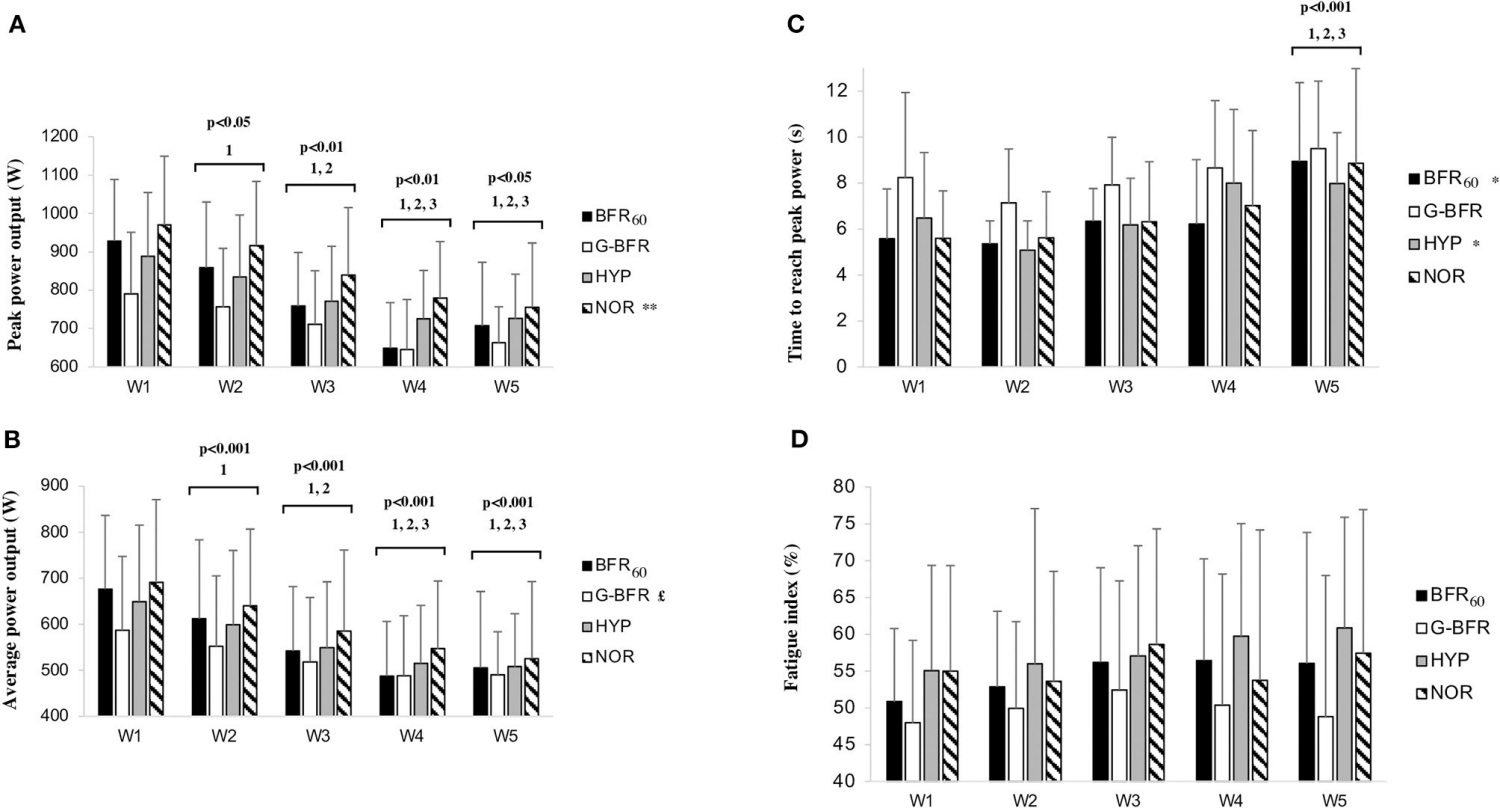
Peak **(A)** and average **(B)** power, time to reach peak power **(C)**, and fatigue index **(D)** between the different conditions. BFR_60_, blood flow restriction during the two first minutes of recovery; HYP, hypoxia in a chamber at 13.0% FiO_2_; G-BFR, gravity-induced BFR; NOR, control. Differences between the numbers of the sprint are highlighted by numbers and symbols are used for differences between conditions. ^1^Different from sprint number one; ^2^different from the second sprint; ^3^different from the third sprint; these differences concern the effect of sprint number without distinguishing the exercise modality (n.b., no statistical interaction was found); *p < 0.05 different from G-BFR; **p < 0.01 different from G-BFR; £*p* < 0.05 different from the other conditions.

A main effect of the condition was observed for peak power (*p* < 0.01) with a lower value found for G-BFR compared to NOR (*p* < 0.01, *d* = −1.0). A main effect of the sprint number was also found for peak power that decreased over time (*p* < 0.01) except between the two last sprints (*p* > 0.05). These results are presented in Figure 2A. The condition had a main effect on average power (*p* < 0.01) and *post-hoc* analyzes showed that values were significantly lower for G-BFR compared to the other conditions (*p* < 0.05, *d* = −0.9, −0.4, and −0.5 for NOR, HYP, and BFR_60_, respectively) (Figure 2B). The sprint number also had an impact on average power as the main effect was detected (*p* < 0.01). Average power decreased over time (*p* < 0.01) except between the two last sprints (*p* > 0.05). Regarding time to achieve peak power, a main effect of the condition was observed (*p* = 0.02). *Post-hoc* analyzes revealed that the time to reach peak power was significantly greater for G-BFR compared to BFR_60_ and HYP (*p* < 0.05, *d* = 1.1, and 1.0, respectively) (Figure 2C). An effect of the sprint number was also detected for the time to achieve peak power (*p* < 0.01) that was longer in the last sprint compared to the first three sprints (*p* < 0.01). Finally, FI did not appear different depending on the condition or the sprint number (*p* > 0.05). These results are shown in Figure 2D.

### Gas Exchanges

Results are presented in Table 1.

**Table 1:** Cardio-respiratory responses during the interventions.

	**BFR_**60**_**	**G-BFR**	**HYP**	**NOR**
**Exercise**				
ΣVT (L)	58.2 ± 15.2	43.7 ± 13.5^‡‡^	61.1 ± 15.1^‡^^**^	61.0 ± 15.9^**^
$\dot{\text{V}}$E_peak_ (L.min^−1^)	150.2 ± 30.7	124.8 ± 27.4^‡‡^	161.9 ± 28.5^‡^^**^	157.7 ± 30.5^**^^$^
$\dot{\text{V}}$E_min_ (L.min^−1^)	39.3 ± 11.9	29.3 ± 11.2^‡‡^	36.0 ± 14.3^**^	38.5 ± 12.4^**^
$\Delta \dot{\text{V}}$E (L.min^−1^)	111.2 ± 24.3	95.5 ± 20.4^‡‡^	125.9 ± 24.7^‡**^	119.2 ± 24.2^**^^$^
ΣVO_2_ (L)	1.208 ± 0.232	0.994 ± 0.297^‡‡^	1.154 ± 0.175^**^	1.243 ± 0.200^**$$^
$\dot{\text{V}}$O_2peak_ (mL.min^−1^)	3872.9 ± 698.8	3895.4 ± 632.3	3422.0 ± 437.8^‡*^	4033.7 ± 543.1^‡^^*^^$^
$\dot{\text{V}}$O_2min_ (mL.min^−1^)	663.9 ± 171.4	562.9 ± 144.3^‡‡^	609.1 ± 199.8	665.5 ± 179.5^**^
$\Delta \dot{\text{V}}$O_2_ (mL.min^−1^)	3202.0 ± 637.8	3332.5 ± 589.5	2812.9 ± 388.8^‡‡**^	3368.2 ± 483.0^‡^^$$^
ΣVCO_2_ (L)	1.106 ± 0.230	0.874 ± 0.193^‡‡^	1.015 ± 0.198^‡‡**^	1.110 ± 0.228^**$$^
$\dot{\text{V}}$CO_2peak_ (mL.min^−1^)	3105.1 ± 828.8	3109.7 ± 713.5	3046.8 ± 734.8	3241.1 ± 830.0^$^
$\dot{\text{V}}$CO_2min_ (mL.min^−1^)	835.3 ± 268.6	675.8 ± 215.7^‡‡^	659.9 ± 229.4^‡‡^	803.3 ± 244.2^**$$^
$\Delta \dot{\text{V}}$CO_2_ (mL.min^−1^)	2269.8 ± 696.4	2433.9 ± 621.6^‡^	2386.9 ± 680.5	2437.8 ± 717.4^‡^
HR (bpm)	138.3 ± 14.3	131.4 ± 14.9^‡‡^	139.9 ± 13.6^**^	142.2 ± 13.5^‡**^
HR_peak_ (bpm)	162.1 ± 11.6	156.4 ± 15.1^‡^	163.0 ± 11.0^**^	165.8 ± 8.5^‡^^**^
HR_min_ (bpm)	113.3 ± 21.2	101.8 ± 20.7^‡‡^	115.8 ± 21.3^**^	117.7 ± 21.2^**^
ΔHR (bpm)	48.8 ± 20.1	54.6 ± 20.5^‡‡^	47.3 ± 18.3^**^	48.0 ± 18.7^**^
**Recovery periods (mean values)**				
ΣVT (L)	281.1 ± 70.8	239.0 ± 67.0^‡‡^	308.4 ± 71.3^‡‡**^	291.9 ± 61.9^**$$^
ΣVO_2_ (L)	5.6 ± 0.9	5.5 ± 0.8	5.9 ± 0.8^‡‡**^	5.9 ± 0.8^‡‡**^
ΣVCO_2_ (L)	6.2 ± 1.5	6.3 ± 1.3	6.3 ± 1.3	6.6 ± 1.3
HR (bpm)	137.1 ± 16.1	123.6 ± 18.6^‡^	139.5 ± 18.1^**^	139.2 ± 16.4^**^
HR_peak_ (bpm)	167 ± 12	164 ± 12	169 ± 12^‡^^**^	171 ± 10^‡‡**^
HR_min_ (bpm)	116 ± 20	104 ± 20^‡^	118 ± 22^**^	119 ± 19^**^
ΔHR (bpm)	51 ± 19	59 ± 16^‡^	50 ± 16^**^	52 ± 15^**^
**Recovery periods**				
**First 2 min**				
ΣVT (L)	166.6 ± 43.9	151.2 ± 39.0^‡‡^	196.4 ± 41.2^‡‡**^	182.4 ± 35.0^‡‡**$$^
ΣVO_2_ (L)	3.512 ± 0.603	3.890 ± 0.520^‡‡^	3.972 ± 0.493^‡‡^	4.029 ± 0.471^‡‡^^*^
ΣVCO_2_ (L)	3.693 ± 1.017	4.231 ± 0.925^‡‡^	4.144 ± 0.870^‡‡^	4.207 ± 1.041^‡‡^
**Last 2 min**				
ΣVT (L)	114.5 ± 28.6	87.8 ± 28.8^‡‡^	112.1 ± 33.7^**^	109.5 ± 28.4^**^
ΣVO_2_ (L)	2.073 ± 0.337	1.633 ± 0.296^‡‡^	1.910 ± 0.347^‡‡**^	1.888 ± 0.336^‡‡**^
ΣVCO_2_ (L)	2.537 ± 0.528	2.065 ± 0.446^‡‡^	2.086 ± 0.434^‡‡^	2.263 ± 0.464^‡‡^^*^^$^

*BFR_60_, blood flow restriction during the two first minutes of recovery; HYP, a hypoxic condition in a chamber (FiO_2_ 13.0%); G-BFR, gravity-induced BFR; NOR, control; ΣVT, cumulated ventilation; $\dot{V}$E_peak_, peak minute ventilation; $\dot{V}$E_min_, minimum minute ventilation; Δ$\dot{V}$E, |$\dot{V}$E_peak_-$\dot{V}$E_min_|; ΣVO_2_, cumulated oxygen consumption; $\dot{V}$O_2peak_, peak oxygen consumption rate; $\dot{V}$O_2min_, mean oxygen consumption rate; $\Delta \dot{V}$O_2_,|$\dot{V}$O_2peak_-$\dot{V}$O_2min_|; ΣVCO_2_, cumulated carbon dioxyde release; $\dot{V}$CO_2_peak, peak carbon dioxyde release rate; $\dot{V}$CO_2_min, minimum carbon dioxyde release rate; $\Delta \dot{V}$CO_2_, |$\dot{V}$CO_2peak_-$\dot{V}$CO_2min_|; HR_mean_, mean heart rate; HR_peak_, peak heart rate; HR_min_, minimum heart rate; ΔHR, |HR_peak_-HR_min_|. Data are expressed as mean ± SD*.

* *Significantly different from G-BFR;*

‡ *significantly different from BFR_60_;*

$ *significantly different from HYP. The statistical significance threshold is set at p < 0.05 (one symbol) and p < 0.01 (two symbols)*.

During sprints, there was a main effect of the condition on ΣVT (*p* < 0.01). Values were lower for G-BFR compared to the other conditions (*p* < 0.01, *d* = −1.2, −1.2, and −1.0 for NOR, HYP, and BFR_60_, respectively), and higher in HYP compared to BFR_60_ (*p* < 0.05, *d* = 0.19). A main effect of the condition was observed for $\dot{\text{V}}$E_peak_ (*p* < 0.01), which was lower in G-BFR compared to the other conditions (*p* < 0.01, *d* = −1.1, −1.3, and −0.9 for NOR, HYP, and BFR_60_, respectively). Values were also lower in BFR_60_ and NOR compared to HYP (*p* < 0.05, *d* = −0.4, and −0.1, respectively).

Oxygen consumption showed a main effect of both conditions (*p* < 0.01) and sprint number (*p* < 0.01). Values were lower in G-BFR compared to the other conditions (*p* < 0.01, *d* = −1.0, −0.7, and −0.8 for NOR, HYP, and BFR_60_, respectively) and in HYP compared to NOR (*p* < 0.01, *d* = −0.5). We found main effects reflecting a difference on $\dot{\text{V}}$O_2peak_ depending on the condition (*p* < 0.01), and the sprint number (*p* < 0.01). Values were lower in HYP compared to all conditions (*p* < 0.05, *d* = −1.2, −0.8, and −0.9 for HYP, BFR_60_, and G-BFR, respectively), and higher in NOR compared to BFR_60_ and G-BFR (*p* < 0.05, *d* = 0.3, and 0.2, respectively). Regarding the sprint number, it was higher in the second sprint compared to the fourth and fifth (*p* < 0.01): 3932 ± 248, 3740 ± 316, and 3656 ± 298 ml.min^−1^, respectively.

Finally, mean sprint heart rate (HR) presented a condition main effect (*p* < 0.01) and a sprint number main effect (*p* < 0.01). HR was lower in G-BFR (*p* < 0.01, *d* = −0.8) and in BFR_60_ (*p* < 0.05, *d* = −0.3) compared to NOR. HR was also lower in the first sprint compared to the other sprints, and in the second sprint compared to the third and the fifth ones (*p* < 0.05): 128.4 ± 5.8, 136.8 ± 5.2, 141.3 ± 5.5, and 143.1 ± 4.4 bpm, respectively. Only a condition main effect was found for HR_peak_ (*p* < 0.01). Values were lower in G-BFR compared to the other conditions (*p* < 0.05, *d* = −0.8, −0.5, and −0.4 for NOR, HYP, and BFR_60_, respectively) and in BFR_60_ compared to NOR (*p* < 0.05, *d* = −0.4).

### Muscular O_2_ Extraction

**Figure 3:**
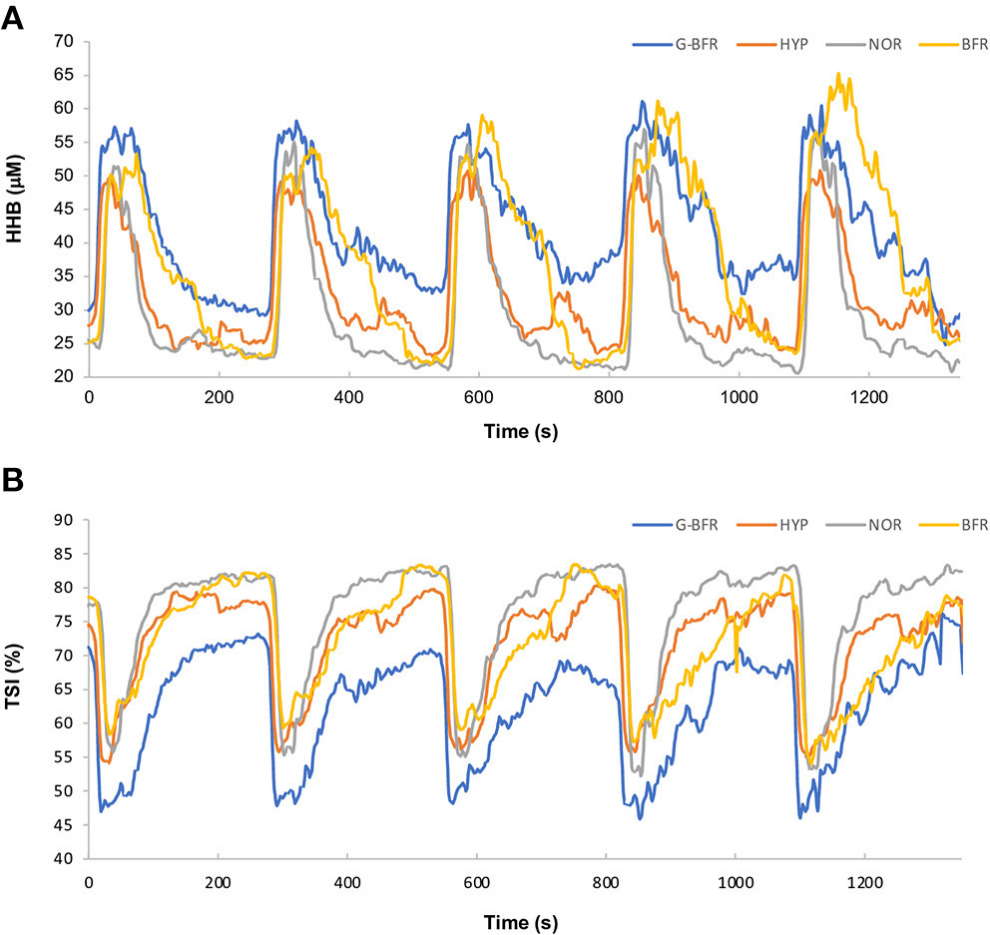
Average deoxyhemoglobin **(A)** and tissue saturation index **(B)**. BFR_60_, blood flow restriction during the two first minutes of recovery; HYP, hypoxia in a chamber at 13.0% FiO_2_; G-BFR, gravity-induced BFR; NOR, control.

Results are presented in Table 2, Figure 3.

**Table 2:** Near-infrared spectroscopy (NIRS) parameters during the interventions.

	**BFR_**60**_**	**G-BFR**	**HYP**	**NOR**
**Exercise**				
TSI (%)	63.7 ± 6.3	54.0 ± 8.2^‡‡^	58.2 ± 8.0^‡‡**^	61.0 ± 6.9^**^
TSI_max_ (%)	80.7 ± 6.4	72.3 ± 10.9	78.8 ± 3.8^**^	80.7 ± 4.6^**^
TSI_min_ (%)	56.0 ± 8.1	46.4 ± 10.0^‡^	49.8 ± 11.1^‡^^*^	52.0 ± 11.2^‡^^*^
ΔTSI (%)	24.7 ± 9.4	25.9 ± 10.5	29.0 ± 11.5	28.7 ± 12.5^‡‡^^*^
[tHb] (μM)	126.6 ± 35.1	105.7 ± 18.4^‡‡^	107.1 ± 22.3^‡‡^	113.2 ± 25.6^‡‡^
[tHb]_max_ (μM)	135.9 ± 38.1	113.5 ± 19.9^‡‡^	115.6 ± 25.8^‡‡^	120.0 ± 27.3^‡‡^
[tHb]_min_ (μM)	120.1 ± 33.8	101.0 ± 17.1^‡‡^	102.3 ± 20.4^‡‡^	107.9 ± 23.8^‡‡^
Δ[tHb] (μM)	15.7 ± 11.5	12.5 ± 4.6	13.2 ± 7.4	12.1 ± 7.2
[O_2_Hb] (μM)	81.0 ± 25.7	56.7 ± 11.3^‡^	61.7 ± 11.7^‡^	68.1 ± 11.7^‡^^*^^$^
[O_2_Hb]_max_ (μM)	108.9 ± 36.5	89.7 ± 21.3^‡^	81.0 ± 19.8^‡^	94.8 ± 21.3^‡^^*^
[O_2_Hb]_min_ (μM)	68.9 ± 23.3	47.0 ± 10.5^‡^	50.9 ± 11.8^‡^	56.2 ± 11.5^‡^^*^
Δ[O_2_Hb] (μM)	40.0 ± 20.6	33.9 ± 13.7	38.8 ± 21.4	38.6 ± 22.5
[HHb] (μM)	45.6 ± 14.4	49.0 ± 14.5	45.4 ± 16.2	45.4 ± 16.2
[HHb]_max_ (μM)	55.6 ± 18.9	57.6 ± 18.9	54.8 ± 20.9	56.5 ± 24.7
[HHb]_min_ (μM)	24.4 ± 8.4	30.2 ± 12.5	23.7 ± 6.6^**^	22.6 ± 8.3^**^
Δ[HHb] (μM)	31.2 ± 18.0	27.4 ± 15.3	31.1 ± 17.0	34.0 ± 21.8^**^
**Recovery periods**				
**First 2 min**				
TSI (%)	72.5 ± 6.6	65.9 ± 10.9	73.7 ± 4.5	77.3 ± 3.0^**^
TSI_max_ (%)	83.4 ± 5.6	78.7 ± 9.2	82.3 ± 3.2	84.4 ± 3.2
TSI_min_ (%)	54.6 ± 8.0	45.7 ± 14.4	50.8 ± 9.8	53.0 ± 7.9
ΔTSI (%)	28.8 ± 9.1	33.0 ± 13.5	31.5 ± 10.3	31.5 ± 8.5
[tHb] (μM)	146.6 ± 52.6	113.2 ± 20.8	113.8 ± 24.4	123.5 ± 30.2
[tHb]_max_ (μM)	178.5 ± 72.7	121.7 ± 24.2^‡^	123.2 ± 29.0^‡^	140.4 ± 46.8
[tHb]_min_ (μM)	111.0 ± 41.0	99.8 ± 20.5	100.7 ± 21.8	105.8 ± 23.0
Δ[tHb] (μM)	67.5 ± 39.6	21.9 ± 11.8^‡^	22.5 ± 9.7^‡^	34.6 ± 30.0^‡^
[O_2_Hb] (μM)	108.5 ± 47.6	74.7 ± 18.2^‡^	84.3 ± 19.7	95.5 ± 23.3
[O_2_Hb]_max_ (μM)	141.0 ± 71.3	92.7 ± 24.2	99.7 ± 26.1	116.4 ± 39.5
[O_2_Hb]_min_ (μM)	67.1 ± 27.1	47.9 ± 14.8	52.4 ± 11.2	58.7 ± 9.3
Δ[O_2_Hb] (μM)	73.9 ± 49.5	44.7 ± 23.7	47.2 ± 21.6	57.7 ± 33.4
[HHb] (μM)	39.1 ± 12.7	39.3 ± 14.2	29.6 ± 7.6	28.0 ± 8.4^‡^
[HHb]_max_ (μM)	61.3 ± 22.1	61.5 ± 24.9	54.9 ± 20.3	56.5 ± 24.7
[HHb]_min_ (μM)	21.8 ± 7.3	24.9 ± 9.2	20.4 ± 4.5	19.8 ± 6.9
Δ[HHb] (μM)	39.5 ± 18.8	36.6 ± 21.2	34.5 ± 17.7	36.6 ± 21.6
**Last 2 min**				
TSI (%)	77.7 ± 6.7	73.0 ± 10.6	79.5 ± 4.1	81.6 ± 3.6^*^
TSI_max_ (%)	84.5 ± 5.1	79.1 ± 8.8	82.3 ± 3.2	84.3 ± 3.5
TSI_min_ (%)	62.7 ± 13.5	63.0 ± 14.5	75.0 ± 6.0^‡^^*^	77.7 ± 6.1^‡^^*^
ΔTSI (%)	21.8 ± 12.0	16.1 ± 10.2	7.3 ± 4.5^‡‡^	6.6 ± 3.9^‡‡^^*^
[tHb] (μM)	136.7 ± 49.7	113.7 ± 20.3	114.5 ± 24.9	123.9 ± 28.0
[tHb]_max_ (μM)	167.2 ± 65.4	120.6 ± 23.6^‡^	121.1 ± 27.4	136.6 ± 41.0
[tHb]_min_ (μM)	112.7 ± 43.9	104.6 ± 21.8	105.8 ± 21.9	111.4 ± 23.2
Δ[tHb] (μM)	54.5 ± 33.9	16.0 ± 12.8^‡‡^	15.4 ± 7.3^‡‡^	25.2 ± 23.9^‡‡^
[O_2_Hb] (μM)	108.4 ± 45.8	83.3 ± 20.2	91.5 ± 22.6	100.8 ± 21.3
[O_2_Hb]_max_ (μM)	136.4 ± 62.3	93.1 ± 23.6	99.0 ± 25.4	113.7 ± 34.8
[O_2_Hb]_min_ (μM)	79.6 ± 42.4	70.9 ± 22.1	82.3 ± 19.2	88.9 ± 16.0
Δ[O_2_Hb] (μM)	56.9 ± 41.1	22.2 ± 16.9^‡^	16.7 ± 9.1^‡‡^	24.8 ± 22.9^‡^
[HHb] (μM)	28.3 ± 11.6	30.4 ± 12.9	23.0 ± 5.7	23.0 ± 8.8
[HHb]_max_ (μM)	45.8 ± 21.6	38.9 ± 19.6	26.5 ± 7.7^‡^	26.3 ± 9.5^‡^
[HHb]_min_ (μM)	19.2 ± 6.6	23.8 ± 9.7	20.2 ± 4.6	20.1 ± 7.8
Δ[HHb] (μM)	26.6 ± 16.5	15.1 ± 12.3^‡^	6.2 ± 4.3^‡‡^	6.2 ± 3.0^‡‡^

*BFR_60_, blood flow restriction during the two first minutes of recovery; HYP, hypoxic condition in a chamber (FiO_2_ 13.0%); G-BFR, gravity-induced BFR; NOR, control; TSI, tissue saturation index; TSI_max_, maximum TSI; TSI_min_, minimum TSI; ΔTSI, |TSI_max_-TSI_min_|; [tHb], total hemoglobin content; [tHb]_max_, maximum [tHb]; [tHb]_min_, minimum [tHb]; Δ[tHb], |[tHb]_max_-[tHb]_min_|; [O_2_Hb], oxyhaemoglobin content; [O_2_Hb]_max_, maximum [O_2_Hb]; [O_2_Hb]_min_, minimum [O_2_Hb]; Δ[O_2_Hb], |[O_2_Hb]_max_-[O_2_Hb]_min_|; [HHb], deoxyhaemoglobin content; [HHb]_max_, maximum [HHb]; [HHb]_min_, minimum [HHb]; Δ[HHb], |[HHb]_max_-[HHb]_min_|. Data are expressed as mean ± SD*.

* *Significantly different from G-BFR;*

‡ *significantly different from BFR_60_;*

$ *significantly different from HYP. Statistical significance threshold is set at p <0.05 (one symbol) and p < 0.01 (two symbols)*.

We found a significant condition main effect on TSI (*p* < 0.01). TSI was higher in BFR_60_ compared to HYP and G-BFR (*p* < 0.01, *d* = 0.8, and 1.3, respectively), and lower in G-BFR compared to the other conditions (*p* < 0.01, *d* = −0.9, −0.5, and −1.3 for NOR, HYP, and BFR_60_, respectively). Mean session (both sprint and recovery periods included) TSI was lower in G-BFR compared to the other conditions (*p* < 0.05). A main effect of condition was detected for [tHb], [tHb]_max_, and [tHb]_min_ (*p* < 0.01). Values were higher in BFR_60_ compared to the other conditions (*p* < 0.01, [tHb]: *d* = 0.4, 0.7, and 0.7; [tHb]_max_: *d* = 0.5, 0.6, and 0.7; [tHb]_min_: *d* = 0.4, 0.6, and 0.7 for NOR, HYP, and G-BFR, respectively).

[O_2_Hb]_max_, [O_2_Hb]_min_, and [O_2_Hb] also presented a main effect of condition (*p* < 0.01). Values were higher in BFR_60_ compared to the other conditions (*p* < 0.05, [O_2_Hb]_max_: *d* = 0.5, 0.7, and 1.0; [O_2_Hb]_min_: *d* = 0.7, 1.0, and 1.2, [O_2_Hb]: *d* = 0.7, 1.0, and 1.2 for NOR, HYP, and G-BFR, respectively), and in NOR compared to G-BFR (*p* < 0.05, [O_2_Hb]_max_: *d* = 0.7; [O_2_Hb]_min_: d = 0.8; [O_2_Hb]: *d* = 1.0). Moreover, [O_2_Hb] was lower in HYP compared to NOR (*p* < 0.05, *d* = −0.5). Finally, an effect of the sprint number was found on Δ[O_2_Hb] (*p* < 0.01). Values increased between the first sprint and the last two sprints (*p* < 0.01): 30.5 ± 2.9, 40.8 ± 6.0, and 42.3 ± 3.9 μM, respectively.

### Recovery Periods

During the first 2 min of recovery, ΣVT, ΣVO_2_, and ΣVCO_2_ presented a main effect of condition (*p* < 0.01). ΣVT was lower in G-BFR (*p* < 0.01, *d* = −0.8, −1.1, and −0.4 for NOR, HYP, and BFR_60_, respectively) and higher in HYP (*p* < 0.01, *d* = 0.4, 0.7, and 1.1 for NOR, BFR_60_, and G-BFR, respectively) compared to the other conditions, and higher in NOR compared to BFR_60_ (*p* < 0.01, *d* = 0.4). During this first part of recovery, ΣVO_2_ was lower in BFR_60_ compared to the other conditions (*p* < 0.01, *d* = −1.0, −0.8, and −0.7 for NOR, HYP, and G-BFR, respectively) and in G-BFR compared to NOR (*p* < 0.05, *d* = −0.3). ΣVCO_2_ was also lower in BFR_60_ compared to the other conditions (*p* < 0.01, *d* = −0.5, −0.5, and −0.6 for NOR, HYP, and G-BFR, respectively). Concerning NIRS data, a main effect of condition was found for TSI (*p* < 0.01). It was lower in G-BFR compared to NOR (*p* < 0.01, *d* = −1.5). Oxyhemoglobin concentration ([O_2_Hb]), deoxyhemoglobin concentration ([HHb]), and the difference of hemoglobin concentration (Δ[tHb]) showed a main effect of condition (*p* < 0.05). [O_2_Hb] was higher for BFR_60_ than G-BFR (*p* < 0.05, *d* = 0.9). [HHb] was higher in BFR_60_ compared to NOR during this period (*p* < 0.05, *d* = 1.1). Δ[tHb] was higher in BFR_60_ compared to the other conditions (*p* < 0.05, *d* = 0.9, 1.6, and 1.5 for NOR, HYP, and G-BFR, respectively).

During the last 2 min of recovery, ΣVT, ΣVO_2_, and ΣVCO_2_ showed a main effect of condition (*p* < 0.01). ΣVT and ΣVO_2_ were lower in G-BFR compared to the other conditions (*p* < 0.01, ΣVT: *d* = −0.8, −0.8, and −0.9, ΣVO_2_: *d* = −0.8; −0.9 and −1.4 for NOR, HYP, and BFR_60_, respectively). Of note, ΣVO_2_ and ΣVCO_2_ were higher in BFR_60_ compared to the other conditions (*p* < 0.01, ΣVO_2_: d = 0.5, 0.5, and 1.4; ΣVCO_2_: *d* = 0.6, 0.9, and 1.0 for NOR, HYP, and G-BFR, respectively). Furthermore, a main effect of condition was observed on TSI, TSI_min_, [tHb]_max_, and [HHb]_max_ (*p* < 0.05). As previously, TSI was lower in G-BFR compared to NOR (*p* < 0.05, *d* = −1.2). Detailed results are presented in Tables 1, 2, Figures 2, 3.

## Discussion

The aim of the present study was to compare the acute effects of systemic hypoxia, BFR_60_, and G-BFR on mechanical output, cardiorespiratory responses, and O_2_ muscle extraction during SIT exercises in healthy moderately trained men. The main results were that SIT associated with G-BFR displayed lower mechanical and cardiorespiratory responses than the other modalities. G-BFR also showed lower skeletal muscle oxygenation. BFR_60_ induced greater blood accumulation within working muscles compared to the other conditions. Moreover, HYP at 13% FiO_2_ and G-BFR increased local hypoxia within the working muscles, with a higher level of hypoxia observed for G-BFR.

The primary results showed that SpO_2_ was lower for HYP throughout the exercise session and decreased at the end of the session (i.e., sprints four and five) for all the conditions. According to Fick's law of diffusion, systemic hypoxemia occurred in HYP because alveolo-capillary oxygen pressure difference decreases under hypoxic conditions. Consequently, $\dot{\text{V}}$E_peak_ increased in HYP, as a compensatory response aiming to maintain oxygen consumption. In accordance, previous studies observed that the magnitude of the ventilatory response appears a critical factor of performance during a Wingate test in hypoxia (Fallon et al., 2015). Indeed, the authors showed that ventilation was elevated at the beginning and throughout the exercise when F_i_O_2_ was decreased from 20.9 to 10% (Fallon et al., 2015). However, the augmentation of ventilation observed in the present study was not sufficient, as total and peak oxygen uptake were still lower in HYP compared to NOR. In addition, mean ventilation was higher in HYP during the first period of recovery. Hypoxemia had an impact on muscle oxygenation parameters. Specifically, oxyhemoglobin concentration during sprints was lower in HYP compared to NOR. Therefore, as total work was the same between the conditions, except for G-BFR, this suggests that the anaerobic metabolism is more solicited in HYP. Furthermore, we analyzed SpO_2_ during recovery when the cuffs were applied and did not observe any difference between the conditions. Similarly, no effect was detected in the study from Willis et al. (2019) using continuous BFR during the RSH protocol at 45% of arterial occlusive pressure.

In the current protocol, fatigue increased progressively throughout the session as performance variables (i.e., peak and mean power, time to reach mean power) were altered, and HR increased with the repetition of sprints. On the other hand, ΣVO_2_ and $\dot{\text{V}}$O_2peak_ were lower during the first sprint probably because metabolic demands are ensured by the anaerobic metabolism (i.e., phosphocreatine hydrolysis and glycolysis). Interestingly, $\dot{\text{V}}$O_2peak_ was higher in NOR compared to the other conditions, which is in accordance with a study from Willis et al. comparing NOR and BFR (Willis et al., 2018). Furthermore, no effect of the condition or the number of sprints was found on FI. A study by Fallon and coworkers (Fallon et al., 2015) observed higher FI in HYP but their study included a single sprint of 30 s with a higher level of hypoxia (FiO_2_ = 10%). In the current study, the BFR_60_ application induced a decrease in gas exchange rates, which increased after its release. This phenomenon has already been observed during partial occlusion in dogs where oxygen consumption was decreased during BFR application and increased when pressure was removed. These responses relied on blood flow, which means that BFR causes vascular resistance (Fales et al., 1962). Importantly, in the current study, BFR was applied for the first 2 min of recovery. This probably delayed and reduced overall recovery, which may influence in turn performance, energy system usage, and metabolite accumulation in the following sprints. The BFR protocol used in the present study was applied during recovery periods between exercise bouts and as such has a different effect to the continuous application that may be seen during other studies. For HYP, performance variables were unaffected but average and maximal oxygen consumption and muscular oxyhemoglobin availability decreased. For all conditions, peak and average power decreased after each sprint excepting between sprints four and five where values were similar. Time to reach peak power increased between the three first sprints and sprint five. RPE increased throughout the exercises. Altogether, these data suggest that during SIT, both BFR and HYP enhance cellular stress (i.e., metabolite accumulation and/or hypoxia) without affecting total work during the training sessions.

Importantly, several cardiorespiratory parameters (i.e., ΣVT, $\dot{\text{V}}$E_peak_, $\dot{\text{V}}$E_min_, Δ$\dot{\text{V}}$E, ΣVO_2_, ΣVCO_2_, HR, HR_peak_, HR_min_, ΔHR), mean TSI (exercise and recoveries), TSI_max_ and TSI_min_ during exercise and/or recovery were lower for G-BFR compared to the other conditions. Of note, during sprints, the supine position induced lower mean, maximal, and minimal tissue saturation. Concerning training data, RPE appeared lower for G-BFR compared to BFR_60_ and HYP, peak power was lower compared to NOR, and time to achieve peak power was higher compared to BFR_60_ and HYP. Average power also appeared lower for G-BFR compared to the other conditions. Overall, these data are consistent with the literature. Indeed, an important decrease in strength of the knee flexor and extensor muscles is inherent to the supine vs. seated position (Houtz et al., 1957). Moreover, maximal exercise performance (such as maximal work rate, $\dot{\text{V}}$O_2max_, $\dot{\text{V}}$E_max_, and HR_max_) during an incremental test has been shown to be impaired in supine position compared to upright cycle position, probably due to a lower perfusion (Hughson et al., 1991). Our results are consistent with the recent study of Preobrazenski and colleagues who showed a decrease in muscle oxygenation in their G-BFR model compared to the control group. However, they reported a higher RPE with G-BFR whereas we obtained the opposite result. This could be explained by the difference in exercise modality (aerobic vs. repeated all-out exercises). Altogether, the G-BFR condition may alter biomechanical and cardiorespiratory responses. However, G-BFR induced lower mean tissue oxygenation. Thus, G-BFR seems to induce specific stress compared to the other modalities.

Concerning BFR_60_ and HYP, present data indicate no difference with NOR for mechanical outcomes. These results are in line with the data from Willis et al. (2019) who compared NOR, BFR, and HYP during an RST protocol. Indeed, the authors found no difference in mean power, mean ventilation, or HR_peak_. They also found a significant increase in total hemoglobin concentration with BFR (constant pressure of 45%). The present results also suggest that BFR_60_ promotes higher blood accumulation within working muscles than the other conditions, meaning that BFR_60_ may additionally affect training adaptations by confining metabolites. Importantly, BFR was suggested to induce greater neuromuscular fatigue while HYP produces central fatigue by impairing corticospinal excitability because of cerebral deoxygenation (Willis et al., 2018; Peyrard et al., 2019). During BFR application, deoxyhemoglobin concentration was higher and oxyhemoglobin content was not different compared to NOR. This result means that a partial occlusion (60%) has a greater impact on venous return than on arterial blood supply. Of note, power output strongly decreases for G-BFR, which may explain in turn the decrease of HR values while it should be increased with higher venous return. Furthermore, enhanced deoxyhemoglobin concentration matches the blood accumulation (increased Δ[tHb]) observed during BFR application. Its release induces bigger changes in oxyhemoglobin, deoxyhemoglobin, and total hemoglobin concentrations compared to the other conditions at the same period. On the contrary, our results suggest that G-BFR does not have a significant impact on venous return, as recently demonstrated by another group during aerobic exercises (Preobrazenski et al., 2021). During sprints, TSI was higher for BFR_60_ compared to the other conditions, due to an increased oxyhemoglobin concentration ([O_2_Hb]). On the other hand, peak and mean HR were lower in BFR_60_ during the exercises. Interestingly, HYP and G-BFR induced greater local hypoxia within skeletal muscles, which was more prominent in G-BFR when considering both exercise bouts and recovery periods. Thus, G-BFR would represent an alternative to HYP to promote additional hypoxic stress within skeletal muscles cells. Additional studies are needed to compare the chronic effects of these conditions on both cellular adaptations to training and gains in muscle performance, especially because G-BFR lowers power output during sessions.

Furthermore, some limits must be acknowledged. First, the modalities of HYP, BFR_60_, and G-BFR conditions were established according to preliminary work that allowed to identify the maximal stress that could be tolerated by the participants in combination with the SIT protocol of this study. Thus, further studies are needed to evaluate more precisely the nature and the degree of the stress generated by these conditions. On the other hand, BFR_60_ was the only stress which was not continuously applied. In such a situation, the BFR application still had a delayed impact on muscle oxygenation parameters during sprints. However, based on preliminary work, this mode of BFR has been chosen to set maximal stress that could be tolerated in this population in combination with the SIT protocol. Another limit can be that participants were moderately trained individuals, and it was a demanding protocol. This also makes it difficult to generalize the results, especially to untrained populations for which these training methods would be hard to complete. Moreover, TSI measurements cover a limited zone of the vastus lateralis muscle thus interpretations of the results at the whole muscle are to be considered with these limitations. Finally, the present study did not include women athletes. Indeed, some adaptations such as cardiovascular responses differ between men and women (Patel et al., 2021), thus introducing variability. Additional works are needed to compare outcomes according to gender because women remain underrepresented in sport science literature.

## Conclusions

In conclusion, this study conducted in healthy moderately trained men showed that a session of SIT in combination with G-BFR showed lower mechanical, cardiorespiratory responses, and skeletal muscle oxygenation than the other conditions. Another important insight is that both SIT protocols conducted under HYP at 13% FiO_2_ and G-BFR amplified local hypoxia within the working muscles. Importantly, a higher level of hypoxia was found with G-BFR when considering the measurement of the entire exercise session (i.e., exercise bouts and recovery periods). Furthermore, a single session of SIT associated with BFR_60_ promotes higher blood accumulation within working muscles compared to the other exercise modes. This suggests that BFR_60_ may additionally or differentially affect cellular homeostasis. Thus, each condition generates specific stress and further studies are needed to better understand subsequent consequences on long-term adaptation.

## Perspectives

Further studies are needed (i) to compare the effects of these protocols on-field performance, (ii) to evaluate more precisely the degree of stress generated by each condition, even if high values for both BFR_60_ and hypoxia have been used based on literature for this kind of exercise, and (iii) to examine mechanistic insight since the mechanisms of action may be different. Investigations of the involvement of different cellular pathways, for example, those involved in mitochondrial adaptations (e.g., the axis of peroxisome proliferator-activated receptor-gamma coactivator 1-alpha) are needed to improve our knowledge about the molecular benefits of these training methods. These research directions are important because they may help to improve the ability to develop more efficient hypoxic or BFR training modalities and to improve skeletal muscle function and whole-body metabolism. Finally, adding stress to training would promote adaptations in the long term, at least when recovery processes are sufficient.

## Data Availability Statement

The raw data supporting the conclusions of this article will be made available by the authors, without undue reservation.

## Ethics Statement

The experimental protocol was approved by the Local Ethics Committee (VD-2021-00597). The patients/participants provided their written informed consent to participate in this study.

## Author Contributions

RS, FB, and AS: conceived and designed the experiments, analyzed the data, designed the tables and figures, wrote the manuscript, contributed reagents, materials, and analysis tools. RS, HB, and FB: performed the experiments. All authors read the manuscript and approved the final version.

## Funding

An internal Bonus Qualité Recherche from the University of Perpignan Via Domitia was obtained to perform this study.

## Conflict of Interest

The authors declare that the research was conducted in the absence of any commercial or financial relationships that could be construed as a potential conflict of interest.

## Publisher's Note

All claims expressed in this article are solely those of the authors and do not necessarily represent those of their affiliated organizations, or those of the publisher, the editors and the reviewers. Any product that may be evaluated in this article, or claim that may be made by its manufacturer, is not guaranteed or endorsed by the publisher.
